# A2BAR Antagonism Decreases the Glomerular Expression and Secretion of Chemoattractants for Monocytes and the Pro-Fibrotic M2 Macrophages Polarization during Diabetic Nephropathy

**DOI:** 10.3390/ijms241310829

**Published:** 2023-06-29

**Authors:** Ángelo Torres-Arévalo, Yéssica Nahuelpán, Katherin Muñoz, Claudia Jara, Claudio Cappelli, Agnieszka Taracha-Wiśniewska, Claudia Quezada-Monrás, Rody San Martín

**Affiliations:** 1 Escuela de Medicina Veterinaria, Facultad de Medicina Veterinaria Y Recursos Naturales, Sede Talca, Universidad Santo Tomás, Talca 347-3620, Chile; 2 Laboratorio de Patología Molecular, Instituto de Bioquímica Y Microbiología, Universidad Austral de Chile, Valdivia 511-0566, Chile; yessica.nahuelpan@alumnos.uach.cl (Y.N.); katherin.munoz@grupobiomedyco.cl (K.M.); claudia.jaracancino@gmail.com (C.J.); claudio.cappelli@uach.cl (C.C.); 3 Faculty of Biology, Institute of Genetics and Biotechnology, University of Warsaw, 02-106 Warsaw, Poland; 4 Tumor Biology Laboratory, Institute of Biochemistry and Microbiology, Faculty of Sciences, Universidad Austral de Chile, Valdivia 511-0566, Chile; claudiaquezada@uach.cl; 5 Millennium Institute on Immunology and Immunotherapy, Universidad Austral de Chile, Valdivia 511-0566, Chile

**Keywords:** adenosine, A2BAR, diabetic nephropathy, macrophages, monocytes, M1/M2, polarization, MRS1754

## Abstract

Some chemoattractants and leukocytes such as M1 and M2 macrophages are known to be involved in the development of glomerulosclerosis during diabetic nephropathy (DN). In the course of diabetes, an altered and defective cellular metabolism leads to the increase in adenosine levels, and thus to changes in the polarity (M1/M2) of macrophages. MRS1754, a selective antagonist of the A2B adenosine receptor (A2BAR), attenuated glomerulosclerosis and decreased macrophage-myofibroblast transition in DN rats. Therefore, we aimed to investigate the effect of MRS1754 on the glomerular expression/secretion of chemoattractants, the intraglomerular infiltration of leukocytes, and macrophage polarity in DN rats. Kidneys/glomeruli of non-diabetic, DN, and MRS1754-treated DN rats were processed for transcriptomic analysis, immunohistopathology, ELISA, and in vitro macrophage migration assays. The transcriptomic analysis identified an upregulation of transcripts and pathways related to the immune system in the glomeruli of DN rats, which was attenuated using MRS1754. The antagonism of the A2BAR decreased glomerular expression/secretion of chemoattractants (CCL2, CCL3, CCL6, and CCL21), the infiltration of macrophages, and their polarization to M2 in DN rats. The in vitro macrophages migration induced by conditioned-medium of DN glomeruli was significantly decreased using neutralizing antibodies against CCL2, CCL3, and CCL21. We concluded that the pharmacological blockade of the A2BAR decreases the transcriptional expression of genes/pathways related to the immune response, protein expression/secretion of chemoattractants, as well as the infiltration of macrophages and their polarization toward the M2 phenotype in the glomeruli of DN rats, suggesting a new mechanism implicated in the antifibrotic effect of MRS1754.

## 1. Introduction

Diabetic nephropathy (DN) is a chronic condition reflected by abnormal changes in the structure and function of the kidneys [1]. It is one of the most common complications of chronic diabetes, affecting over 40% of diabetic patients and remaining one of the major causes of end-stage renal disease (ESRD) in most countries of the world [2,3]. The pathogenesis of the development and progression of DN is complex and multifactorial, with several pathological pathways leading to the histological damage observed in renal biopsies of DN patients [4]. Characteristic histological changes in the glomerulus during DN include thickening of the glomerular basement membrane and mesangial matrix expansion, occurring with or without nodular sclerosis, referred to as Kimmelstiel–Wilson lesions [5]. As the disease progresses, patients develop interstitial fibrosis with tubular atrophy along with arteriolar hyalinosis. Changes in cell architecture include podocyte loss and endothelial disruption, which ultimately lead to nephron loss [5,6,7,8,9]. The loss of glomerular function manifests itself clinically by the presence of excess proteins in the urine (proteinuria) and/or decline of the glomerular filtration rate (GFR) [7]. Patients with DN who progress to chronic kidney disease (CKD) exhibit an increase in urine production (polyuria), the appearance of glucose in the urine (glycosuria), and elevated blood urea nitrogen (BUN) and serum creatinine levels [10]. Current standard DN therapy consisting of antihypertensive, antidyslipidemic, and new anti-hyperglycemic agents, such as sodium-glucose cotransporter inhibitors (SGLT2i) and glucagon-like peptide 1 receptor agonists (GLP1-RA), have been shown to slow the progression of the renal damage and reduce DN-related mortality [11,12]. Despite advances, treatment remains a challenge requiring urgent solutions and further research.

Although clinical and experimental data demonstrate that DN is not a primary immune-mediated disease, an increasing body of evidence supports an important role for the immune system in its development and progression [13,14]. Several elements of the immune system, including cytokines and resident chemokines, recruitment of neutrophils, monocytes, macrophages (MΦs), and T lymphocytes, as well as the deposition of the immune complex, are known to be associated with the condition, and appear in the diabetic kidney as a response to progressive renal damage [13,15,16,17]. IL-1β, IL-6, TNF-α (Tumor Necrosis Factor-α), IL-8, MIP-1α/CCL2 (Macrophage Inflammatory Protein-1α/Chemokine C-C motif ligand 2). RANTES/CCL5 (Regulated on Activation, Normal T cell Expressed, and Secreted/Chemokine C-C motif ligand 5) are among the most relevant chemokines and cytokines involved in in chemoattraction and infiltration of immune cells (leukocytes) into the kidney, contributing to the development of DN [18,19,20].

As monocytes/MΦs infiltrate and accumulate in the tubule-interstitium and the glomerulus [21], they transition to myofibroblasts (α-SMA+, Col1+, Fn-1+) in a process known as macrophage–myofibroblast transition (MMT) [22,23,24], leading to massive depositions of extracellular matrix (ECM) and kidney dysfunction [25]. In addition, due to their great functional plasticity known as polarization, MΦs have been classified into classical M1 (CD86+, CD38+, CD11c+, iNOS+, CD80+, and CCR7+) and alternatively activated M2 (CD163+, Egr2+, CD86-, CD163+, and CD206+) with proinflammatory and anti-inflammatory/profibrotic characteristics, respectively [26,27]. Both phenotypes participate in the progression of fibrosis, with excess activation of M1 MΦs leading to the death of renal resident cells and altering the proper tissue repair; meanwhile, M2 MΦs promote a fibrotic response and tissue repair, which can become aberrant and dysregulated in renal fibrosis [28].

Interestingly, it has been documented that DN progresses with growing levels of the nucleoside adenosine [29,30]. Adenosine is a potent autocrine anti-inflammatory and immunosuppressive molecule, the receptors of which are known to mediate immunomodulatory actions, contributing to tissue repair [31], and it plays a substantial role in renal physiology [32]. Activation of the A2B adenosine receptor (A2BAR) in the glomerulus causes an increased release of VEGF (Vascular Endothelial Growth Factor) and TGF-β (Transforming Growth Factor beta), which is conducive to the progression of fibrosis during DN [33,34]. Studies on DN rats treated with a selective A2BAR antagonist, MRS1754, showed an attenuation of some of the clinical and histological signs of glomerulosclerosis, as well as decreased intraglomerular MΦs infiltration and MMT [35]. Additionally, other selective A2BAR antagonist, PSB1115, inhibited the expression of chemokines (MCP-1/CCL2 and RANTES/CCL5), inflammatory mediators (IL-1β and IL-6), and fibrotic factors (TGF-β and Collagen I), and decreased the proteinuria and the infiltration/activation of M1 MΦs in the early stage of renal injury in a unilateral ureteral obstruction (UUO) mouse model of CKD [36]. Despite all the advances in our understanding of the disease, the contribution of the A2BAR to the glomerular chemoattraction and infiltration of leukocytes and MΦ polarity during DN has not been studied in depth. Here, we aimed to examine the effect of the pharmacological antagonism of the A2BAR on the expression and secretion of chemokines/chemoattractants for immune cells, intraglomerular infiltration of leukocytes, and MΦ polarity in rats with DN.

## 2. Results

### 2.1. The In Vivo Blockade of the A2BAR Alters the Expression of Immune System-Related Genes in the Glomeruli of Diabetic Nephropathy Rats

To validate our model, we evaluated proteinuria, glomerulosclerosis, and podocyte markers in DN rats treated with MRS1754 (Figure S1). Then, we isolated glomeruli from healthy non-diabetic (control, Ctrl), DN, and DN + MRS1754 rats and performed RNA-seq studies. The bioinformatic analysis revealed an upregulation of pathways related to immune system in the glomeruli of DN rats (Table 1). In turn, downregulated pathways in the glomeruli of DN rats were not related to immune response. Among the upregulated pathways, the most relevant were related to Interleukin-33 signaling, chemokine receptors/chemokines, Interleukin-10 signaling, Interleukin receptor SHC signaling, Terminal pathway of complement, Peptide ligand-binding receptors, and CASP8 activity inhibition (Table 1). These data suggests that the expression of immune-related genes and pathways is upregulated in the glomeruli of DN rats.

**Figure 1:**
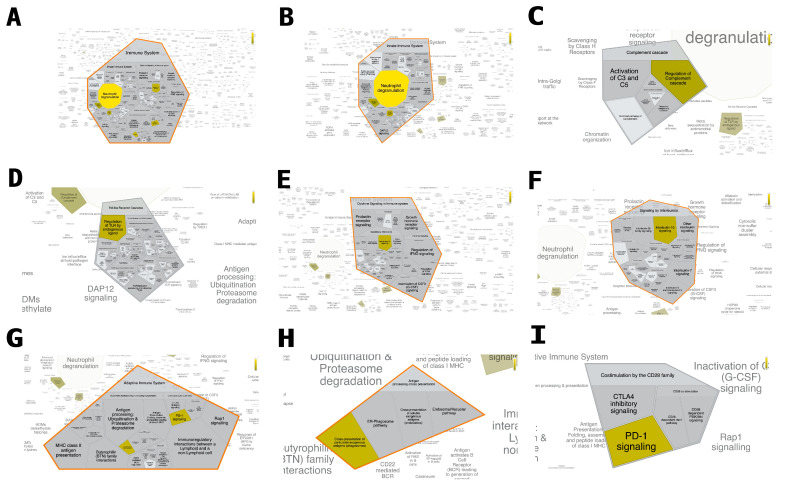
Genome-wide overview of the immune system related-transcripts in the glomeruli of DN + MRS1754 rats. Reactome pathways are arranged in a hierarchy. Analysis of pathways related to (**A**) Immune System, (**B**) Innate Immune System, (**C**) Complement cascade, (**D**) Toll-like Receptor Cascades, (**E**) Cytokine Signaling in Immune system, (**F**) Signaling by Interleukins, (**G**), Adaptative Immune System (**H**) Antigen processing-Cross presentation, and (**I**) Coestimulation by the CD28 family in transcripts of glomeruli isolated from DN + MRS1754 rats. The color code denotes over-representation of that pathway in the input dataset. Light grey signifies pathways which are not significantly over-represented. Analysis was performed using https://reactome.org (accessed on 5 June 2023).

MRS1754 treatment dysregulated the transcriptional expression of signaling pathways related to immune system regulation in the glomeruli of DN rats (Figure 1 and Figure S2). A total of 394 of 754 transcripts dysregulated in the DN + MRS1754 glomeruli were related to the immune system (Tables S1 and S2). Moreover, out of 664 transcripts identifiers in the sample, 490 were represented in Reactome, with 1638 pathways found to be hit by at least one of them. A total of 13 of the 25 most relevant pathways downregulated in the DN + MRS1754 glomeruli, sorted by *p*-value, were related to the immune response (Table 2). Upregulated pathways were not related to immune system in the glomeruli of DN + MRS1754 rats. KEGG analysis indicated that 42 out of 81 dysregulated signaling pathways analyzed are implicated in the immune response (Table S3). The genes involved in both innate and adaptative immune response were dysregulated in DN + MRS1754 glomeruli. Among the downregulated pathways, the most relevant were related to neutrophil degranulation, Neutrophil degranulation, PD-1 signaling, IRAK deficiency, Interleukin-10 signaling, RUNX3 regulates immune response and cell migration, MyD88 deficiency, translocation of ZAP-70 to immunological synapse, cross-presentation of particulate exogenous antigens, immunoregulatory interactions between a lymphoid and a non-Lymphoid cell, phosphorylation of CD3 and TCR zeta chains, activation and regulation of complement cascade, chemokine receptors/chemokines, and trafficking and processing of endosomal TLR (Table 2). This information suggests that MRS1754 treatment decreases the expression of immune-related genes and pathways in the glomeruli of DN rats.

### 2.2. MRS1754 In Vivo Treatment Decreases the Expression and Secretion of Chemoattractants/Chemokines for Immune Cells in Rats with Diabetic Nephropathy

**Figure 2:**
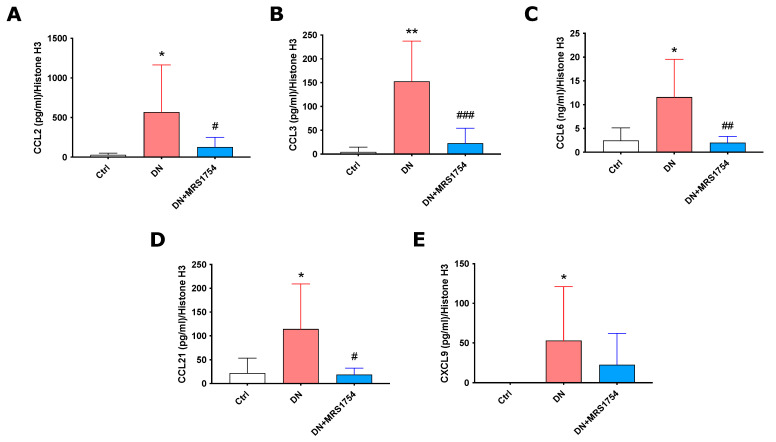
Chemokines/chemoattractans secreted by glomeruli of DN + MRS1754 rats. Quantification of (**A**) CCL2, (**B**) CCL3, (**C**) CCL6, (**D**) CCL21, and (**E**) CXCL9 by ELISA in the CM of glomeruli isolated from Ctrl, DN, and DN + MRS1754 rats. Measures of each chemokine/chemoattractant were normalized by H3 quantification by western blot (Figure S1). Graphs represent distribution of each sample and mean ± S.D. * *p* < 0.05, and ** *p* < 0.01 versus Ctrl rats. ^#^ *p* < 0.05, ^##^ *p* < 0.01, ^###^ *p* < 0.001 DN + MRS1754 versus DN rats. *n* = 6 samples per duplicate.

Previously, we demonstrated that the in vivo administration of MRS1754 decreases the transcripts of chemokines/chemoattractants (CCL2, CCL3, CCL6, CXCL9, and CCL21) for leukocytes in the glomeruli of DN rats [35]. To determine the effect of the antagonism of the A2BAR on the secretion of these chemokines in the glomerulus, ELISA assays were performed with the conditioned medium (CM) of ex vivo glomeruli isolated from Ctrl, DN, and DN + MRS1754 rats. CCL2, CCL3, CCL6, CCL21, and CXCL9 were present in higher concentrations in the CM of ex vivo glomeruli from DN rats (Figure 2). In vivo treatment with MRS1754 decreased the concentration of the chemokines CCL2, CCL3, CCL6, and CCL21 in the CM of DN glomeruli (Figure 2).

**Figure 3:**
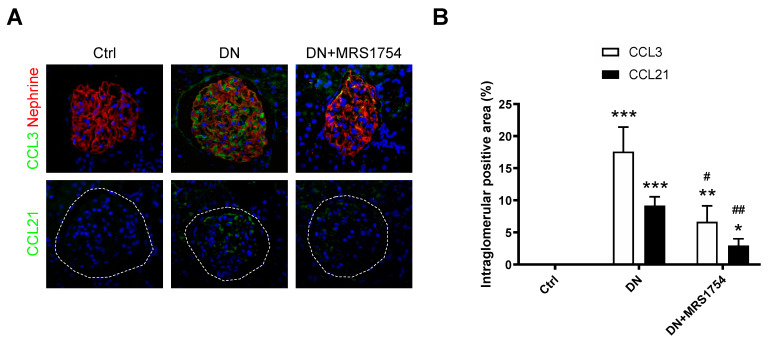
Expression of chemokines/chemoattractans in glomeruli of DN + MRS1754 rats. (**A**) Protein expression of CCL3 and CCL21 (green color) by immunohistofluorescence in glomeruli (red color) of Ctrl, DN, and DN + MRS1754 rats. The dotted lines represent glomeruli areas. (**B**) Quantification of positive intraglomerular area (%) of (**A**). Graphs represent the mean ± S.D. * *p* < 0.05, ** *p* < 0.01 and *** *p* < 0.001 versus Ctrl rats. ^#^ *p* < 0.05 and ^##^ *p* < 0.01, DN + MRS1754 versus DN rats. *n* = 4.

Since the transcriptional expression and the secretion of CCL3 and CCL21 underwent robust changes in the glomeruli from DN + MRS1754 rats, the differences in their protein expression at the glomerular level were evaluated through immunohistofluorescence (IHF). The expression of CCL3 and CCL21 was not detected in the glomeruli of Ctrl rats, but was present in DN rats where the positive intraglomerular area for both chemokines reaches 17.6% and 9.2%, respectively (Figure 3). The in vivo pharmacological blockade of A2BAR in DN rats decreased the positive area of expression in the glomeruli to 6.6% and 2.9% for CCL3 and CCL21, respectively (Figure 3). These results suggest that the in vivo administration of MRS1754 decreases the glomerular protein expression and secretion of chemoattractants/chemokines for immune cells in DN rats.

### 2.3. MRS1754 In Vivo Treatment Decreases the Intraglomerular Infiltration of Monocytes/Macrophages through the Reduction of Chemoattractant/Chemokine Expression/Secretion in Rats with Diabetic Nephropathy

**Figure 4:**
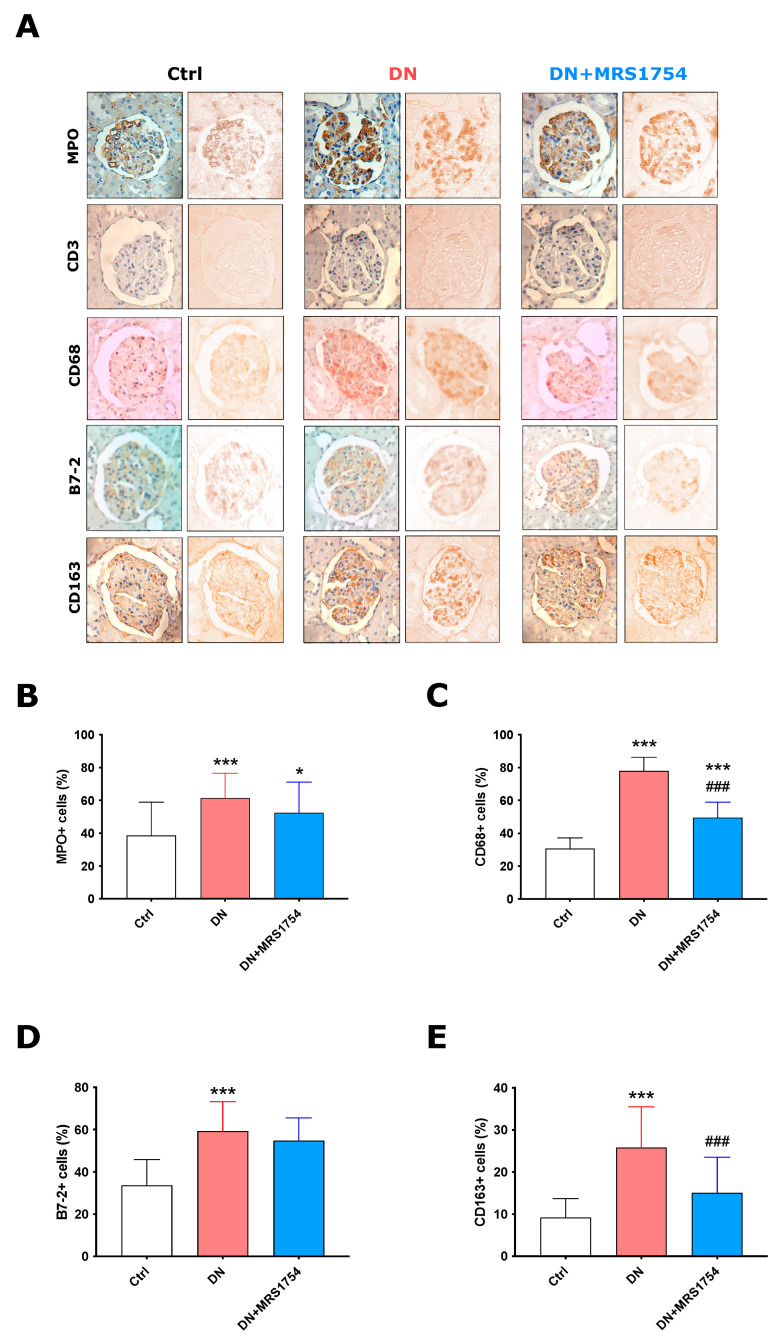
Infiltration of leukocytes in the glomeruli of DN + MRS1754 rats. (**A**) Infiltration of neutrophils (MPO+), T lymphocytes (CD3+), monocytes/MΦs (CD68+), M1 (B7-2+), and M2 (CD163+) MΦs by immunohistochemestry in the glomeruli of Ctrl, DN, and DN + MRS1754 rats. The images on the left correspond to the original capture, and the images on the right correspond to the deconvoluted color channel of the DAB stain obtained through ImageJ analysis. The images show the areas specific for glomeruli with the maginification of 400×. (**B**–**E**) Quantification of positive intraglomerular area (%) of (**B**) neutrophils (MPO+), (**C**) monocytes/MΦs (CD68+), (**D**) M1 MΦs (B7-2+), (**E**) M2 MΦs (CD163+). Graphs represent distribution of each sample and the mean ± S.D. * *p* < 0.05, and *** *p* < 0.001 versus Ctrl rats. ^###^ *p* < 0.001, DN + MRS1754 versus DN rats. *n* = 4.

In addition, we evaluated the intraglomerular infiltration of leukocytes, such as neutrophils, T lymphocytes, and monocytes/MΦs, which are targets of the evaluated chemoattractants/chemokines (Figure 4A). The percentage of intraglomerular positive area for neutrophils (MPO+) and monocytes/MΦs (CD68+) markers was found to be 1.58 and 2.53 times higher, respectively, in DN rats compared to the control group (Figure 4A–C). The infiltration of T lymphocytes (CD3+) was not detected in the glomeruli of Ctrl, DN, and DN + MRS1754 rats (Figure 4A). Interestingly, the pharmacological blockade of the A2BAR only diminished the infiltration of monocytes/MΦs 1.57-fold, but did not affect the infiltration of neutrophils and lymphocytes (Figure 4A,C).

**Figure 5:**
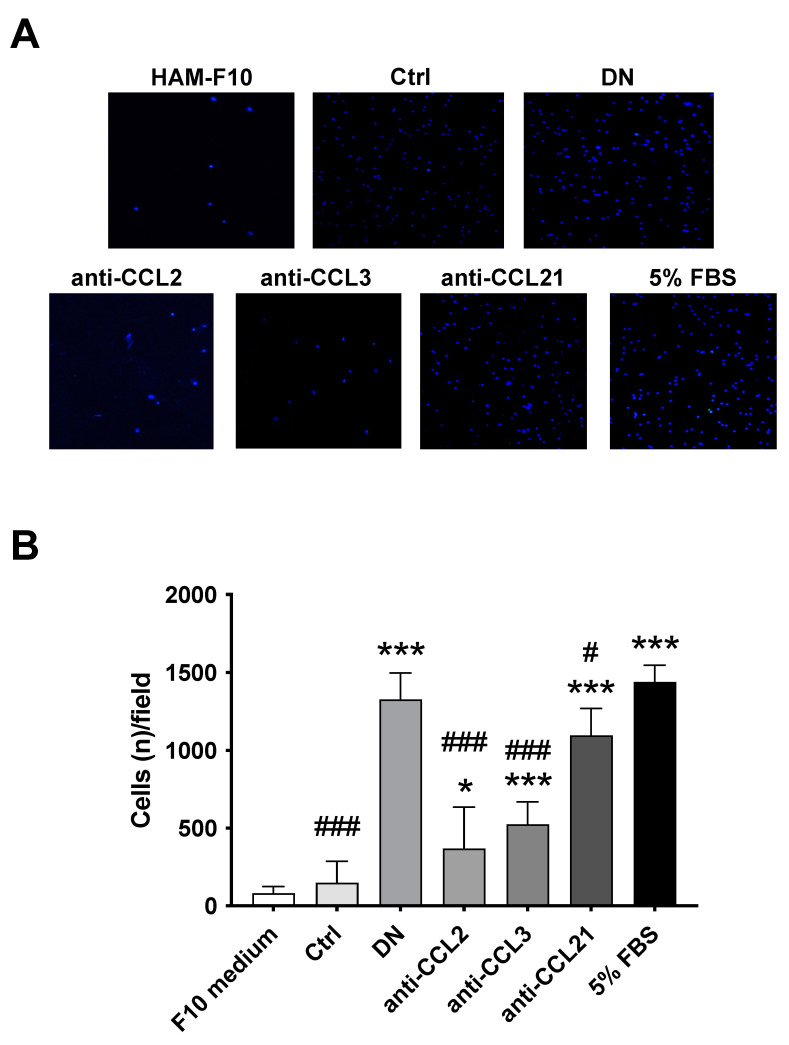
Effect of neutralizing antibodies for chemokines/chemoattractans in monocytes/MΦs in vitro cell migration assays. (**A**) The effect of the neutralizing antibodies against CCL2 (500 ng/mL), CCL3 (500 ng/mL), and CCL21 (500 ng/mL) in the monocytes/MΦs migration assay under the chemoattractant effect of the CM of the glomeruli from DN rats. HAM-F10 and 5% FBS were used as negative and positive control, respectively, of in vitro stimuli for migration. DAPI was used to stain the nuclei of monocytes/MΦs. Representative quadrants show monocytes/MΦs in blue (400× magnification). (**B**) Quantification of positive intraglomerular area (%) of deconvoluted color for DAB in (**A**). Graphs represent distribution of each sample and the mean ± S.D. * *p* < 0.05, and *** *p* < 0.001 versus Ctrl rats. ^#^ *p* < 0.05 and ^###^ *p* < 0.001, DN + MRS1754 versus DN rats. *n* = 4.

Given that CCL2, CCL3, and CCL21 are chemoattractants of monocytes/MΦs [37], and their regulation was affected by A2BAR antagonism, we used neutralizing antibodies in an in vitro assay to determine the chemoattracting effect of each chemokine in the monocytes/MΦs migration under the stimuli of the glomeruli CM from DN rats (Figure 5). The in vitro monocytes/MΦs migration increased 8.8-fold with the CM of the glomeruli from DN rats, which was restored 3.96, 2.5, and 1.14-fold using neutralizing antibodies for CCL2, CCL3, and CCL21, respectively (Figure 5). These data suggests that the in vivo antagonism of A2BAR decreases the infiltration of monocytes/MΦs in the glomeruli of DN rats through the reduction of the expression/secretion of chemoattractants/chemokines.

### 2.4. The In Vivo Antagonism of the A2BAR Decreased M2 Macrophage Polarization in the Glomeruli of Rats with Diabetic Nephropathy

When monocytes infiltrate the kidney, they differentiate into M1 (B7-2+) or proinflammatory MΦs, which under a profibrotic microenvironment polarize to M2 (CD163+) MΦs. We observed an increase in 76.3% and 80.1% percent in the number of M1 and M2 MΦs, respectively, in the glomeruli of diabetic rats compared to those of the control group (Figure 4). MRS1754 treatment did not affect the infiltration of M1 MΦs; however, it decreased the number of polarized M2 MΦs by 71.26% in the glomeruli of DN rats (Figure 4A,D,E). In this way, the in vivo antagonism of A2BAR with MRS1754 diminishes the intraglomerular M2 MΦ polarization during DN in rats.

## 3. Discussion

We demonstrated that the in vivo antagonism of the A2BAR diminished the glomerular transcripts and protein expression and secretion of chemokines/chemoattractants, decreasing the intraglomerular infiltration of monocytes and pro-fibrotic M2 MΦ polarization during DN. Previous studies have shown the antifibrotic effects of MRS1754 by the regulation of VEGF and TGF-β secretion [33,34,38]. However, in this study, we introduce a novel mechanism that explains the role of adenosine and its receptor, A2BAR, on glomerulosclerosis through the regulation of the infiltration of MΦs and the pro-fibrotic M2 MΦ polarization in the glomeruli of DN rats. These results suggest that the use of A2BAR antagonists could become part of the treatment of DN in humans. Many novel drugs, the mechanism of action of which is based on the antagonism of adenosine receptors, in particular A2BAR, are currently in clinical trials for the treatment of various pathologies, including idiopathic pulmonary fibrosis and cancer [39,40,41]. Approving human use of any of these drugs would likely contribute to evaluating their effectiveness in other pathologies such as DN.

To evaluate the effect of the antagonism of A2BAR on the glomerulus during diabetes we used the rat STZ-induced DN model [42] to perform transcriptomic and bioinformatic analyses. We found that the in vivo administration of MRS1754 for eight weeks downregulated the transcriptional expression of genes and pathways related to the immune system in the glomeruli of DN rats. We have previously shown that MRS1754 administered using the same regiment of doses and duration, dysregulated transcripts related to focal adhesion/cell adhesion molecules and the chemokine signaling pathway/leukocyte transendothelial migration in the glomeruli of DN + MRS1754 rats [35].

Our results expose that the protein expression and secretion of MCP-1/CCL2 (Monocyte chemoattractant protein-1/Chemokine C-C motif ligand 2), MIP1-α/CCL3 (Macrophage inflammatory protein 1-alpha/Chemokine C-C motif ligand 3), CCL6 (Chemokine C-C motif ligand 6), SLC/CCL21 (Secondary lymphoid tissue chemokine/Chemokine C-C motif ligand 21), and MIG/CXCL9 (Monokine induced by gamma interferon/Chemokine C-X-C motif ligand 9) increased in the glomeruli of DN rats. Different studies have validated the effect of adenosine and the activation of its receptors as a chemotaxis signal for leukocytes [43,44,45]. In the present study, we demonstrate that MRS1754 decreased the protein expression and secretion of chemokines attractants for immune cells such as CCL2, CCL3, CCL6, and CCL21 [37]. CCL2 is the main chemokine driving leukocyte infiltration and monocyte recruitment during acute and chronic inflammatory response in the kidney disease [46,47,48,49,50]. As expected, the protein expression and secretion of CCL2 was higher in DN glomeruli compared to Ctrl rats. Surprisingly, the in vivo blockade of A2BAR decreased the protein expression/secretion of CCL2 in the glomeruli of DN rats, which could explain the lower infiltration of monocytes/MΦs in DN + MRS1754 compared to DN rats. CCL3, a member of the CC chemokine family, plays an important role in the development, regulation, and recruitment of leukocytes [37,51]. CCL3 is produced by a variety of cells, including lymphocytes, fibroblasts, and epithelial cells [52,53,54,55]. This chemokine has been reported to be chemotactic for both neutrophils and monocytes in mice in vitro and in vivo [52,56,57]. This could explain the increased infiltration of MPO+ (neutrophils) and CD68+ (monocytes/MΦs) cells in DN rats. CCL6 is a small chemokine only reported in rodents, which is mainly produced in monocytes, MΦs [58], and eosinophils during allergic airway inflammation [59]. Overexpression of CCL6 has been associated with tumor growth and metastatic spread in mice [60]. While the expression and function of CCL6 in the kidney has not been completely characterized, some investigations have been linking it to renal inflammatory processes and an ischemia reperfusion injury-induced kidney fibrosis model in mice [61,62]. Here, we present for the first time that CCL6 is implicated in DN and the extracellular adenosine axis; however, the levels of secretion of this chemokine were lower than the other chemoattractants evaluated. More studies are necessary to determine how adenosine and its receptor, A2BAR, are involved in the control of the expression and secretion of CCL6 during DN. CCL21 is a member of the CC chemokine family, which is a potent chemoattractant for C-C chemokine receptor type 7 positive (CCR7+) cells, such as T cells, B cells, dendritic cells, fibrocytes, and M1 MΦs [63,64,65,66,67,68,69,70]. Nevertheless, in this study, the impact of the antagonism of A2BAR seems to be focused on monocytes/MΦs and not on the other cells, since MRS1754 has only decreased the intraglomerular infiltration of these cells. Nonetheless, further investigation should be carried out to evaluate the effect of MRS1754 in the infiltration of B cells, dendritic cells, and fibrocytes. On the other hand, the effect of MRS1754 on the intraglomerular monocytes/MΦs infiltration may be direct, as these cells express high levels of A2BAR supporting the metabolism of MΦs and enabling them to persist in the tissue [44,71,72]. To corroborate this, it would be necessary to use another in vivo model such as adenosine receptor knockout (KO) animals, lacking the receptor specifically in monocytes/MΦs [73,74].

Because of their great heterogeneity, MΦs participate in various cellular events, including phagocytosis, presentation of external soluble antigens, and regulation of tissue fibrosis [26,27,75,76]. This functional plasticity, known as polarization, is a consequence of specific biological functional phenotypes acquired in response to different microenvironmental stimuli. Typically, this polarization has allowed MΦs to be classified into classical M1 (CD38+, CD11c+, iNOS+/CD80+/CCR7+) and alternatively activated M2 (Egr2+, CD86−/CD163+/CD206+) MΦs, having pro-inflammatory and anti-inflammatory/pro-fibrotic properties, respectively [26,27,75]. Both phenotypes participate in the progression of DN [77,78]. A growing and important number of investigations have shown that the function of the immune system is altered in DM, with hyperglycemia being one of the factors responsible for this dysfunction [79,80,81,82,83,84]. Hyperglycemia can modify the activity of various leukocytes, including monocytes/MΦs [85,86]. Both in vitro and in vivo studies indicate that chronic hyperglycemia increased polarization of M2 MΦs in diabetic mice and in bone marrow-derived MΦs exposed to high glucose concentrations [79,87,88], which would contribute to the progression of tissue fibrosis in DM. During tissue repair, MΦs remodel the ECM and phagocytose apoptotic cells, but these processes are dysregulated in DM [89,90]. In this study, we observed an increase in M1 and M2 MΦs in the glomeruli of DN rats, which supports the idea that both MΦs participate during the pathophysiology of glomerulosclerosis [77,78]. Adenosine is capable of regulating the activation of M1 and M2 MΦs through the A2AAR and A2BAR, respectively [72,91]. Activation of A2AAR on M1 MΦ suppresses the production of cytokines, such as TNF-α, IL-12, and nitric oxide, thus exerting an anti-inflammatory effect [72]. On the other hand, the activation of A2BAR increases the expression of the enzymes arginase-1 and TIMP-1 and enhances the differentiation of M2 MΦs induced by IL-4 and IL-12 [91], thus contributing to tissue repair and fibrosis. The A2BAR appears to be involved in the intraglomerular infiltration and myofibroblastic differentiation of MΦs during DN. In vivo antagonism of A2BAR using MRS1754 decreases the transcription of monocyte chemoattractant molecules and the myofibroblastic transition of MΦs, thereby attenuating glomerular fibrosis [35]. Although adenosine and its A2BAR regulate the polarization of MΦs, the decreased number of M2 MΦs in the glomeruli of DN MRS1754-treated rats could be attributed to diminished monocytes/MΦs availability, likely resulting from the reduced infiltration of these cells. These results and data show the effect of adenosine and A2BAR on the glomerular infiltration, polarization, and fibrotic function of monocytes/MΦs during DN. In the future, we intend to evaluate the effect of the leukocyte recruitment, in particular M1/M2 MΦs, in the tubuli of diabetic rats with tubulointerstitial renal fibrosis treated with MRS1754.

## 4. Materials and Methods

### 4.1. Animals and Sample Biopsies

One-month-old male Sprague-Dawley rats, weighing 200–250 g, received a single intravenous injection of streptozotocin at a dose of 65 mg/kg (STZ; Merck, Darmstadt, Germany) to induce diabetes mellitus (DM) [92]. Rats from the control group (non-diabetic; Ctrl) were inoculated with an equivalent volume of STZ vehicle (citrate buffer pH 4.5). One week after the injection, blood glucose levels were measured and DN was confirmed when glycemia levels between 300–500 mg/dL, occurring along with proteinuria and glucosuria, were observed. Four weeks after the STZ injection, DN rats were treated with intraperitoneal injections of MRS1754 (DN + MRS1754; 0.5 mg/kg/48 h; Tocris Bioscience, Bristol, UK) [33,35] or an equivalent volume of MRS1754 vehicle (DN), 1X phosphate buffered saline (1X PBS) over a period of eight weeks. Glycemia and body weight were measured weekly. At week twelve post-STZ inoculation, all rats (16-weeks old) were euthanized using an overdose of an inhalational anesthetic (Isoflurane #10019036060; Baxter, Deerfield, IL, USA). Kidneys were then removed, placed in ice-cold 1X PBS, and stored in this same solution until further analyses and histological processing. All procedures were carried out in accordance with the National Institutes of Health Guide for the Care and Use of Laboratory Animals, 8th edition (2011); http://grants.nih.gov/grants/olaw/guide-for-the-care-and-use-of-laboratory-animals.pdf (accessed on 21 July 2022) and approved by the Institutional Committee on the Use of Live Animals in Research at the Universidad Austral de Chile (approval number 309/2018). All possible measures were taken to minimize animal suffering and to reduce the number of animals used.

### 4.2. Glomerulus Isolation

To isolate the glomeruli, kidneys were washed in 1X PBS, chopped, and gradually sieved through 212, 150, 106, and 75-μm meshes. The material was then collected and centrifuged at 1500× *g* for 5 min at room temperature, yielding a fraction of the glomeruli with a purity ≥ 90% [92]. This fraction was used for transcriptomic analyses, and in vitro rat MΦ migration assays.

### 4.3. Transcriptomic Analysis

The transcriptomic analysis was performed as previously described [35]. Briefly, glomerular RNA from DN and DN + MRS1754 rats was isolated with the use of the commercial kit Nucleospin RNA II (Macherey-Nagel, Duren, Germany) following the instructions specified by the manufacturer. Samples were analyzed on an Advanced Analytical Fragment Analyzer (2100 Bioanalyzer, G2939BA Agilent, Santa Clara, CA, USA) to assess RNA quality and assign an RQN (RNA Quality Number) score. RQN values greater than or equal to 8 were considered suitable for library preparation. The RNA-Seq library was generated using the TruSeq RNA Sample Preparation Kit (Illumina) and quantified through quantitative real-time PCR (qPCR) with the Library Quant Kit Illumina GA (KAPA), following the manufacturer’s instructions. Libraries were clustered on-board and sequenced to generate 125 bp paired-end reads using the high-throughput sequencing system HiSeq2500 (Illumina). Sequences were mapped to the rat genome (ensembl.org) and the number of read counts per gene was determined for each library using the feature counts function of the Rsubread R library. To determine differential expression based on raw counts we used the DEseq2 R library, where a *p*-adjusted value equal or less than 0.05 was considered statistically significant. Transcripts with significant statistical differences (*p* ≤ 0.05) were subjected to Reactome, Database for Annotation, Visualization and Integrated Discovery (DAVID) v6.8, and Kyoto Encyclopedia of Genes and Genomes (KEGG) [93,94].

### 4.4. Immunohistochemistry and Immunohistofluorescence

Kidneys from Ctrl, DN, and DN + MRS1754 rats were extracted, fixed in 4% paraformaldehyde (PFA), paraffin embedded, and sectioned. After mounting on silanized slides, the 5-μm-thick sections were deparaffinized with xylene and rehydrated in a series of solutions of decreasing ethanol concentration (96%, 90%, 70%, and 50% ethanol). Heat-mediated antigen retrieval was performed in citrate buffer (10 mM sodium citrate, 0.05% Tween 20, pH 6.0) for 20 min using a pressure cooker (BioSB TintoRetriever). Subsequently, samples were cooled at room temperature for 30 min. For immunohistochemistry (IHC), samples were incubated with 3% H2O2 for 10 min. For blocking, 2.5% normal horse serum (S-2012-50; Vector Laboratories, Newark, CA, USA) and 1% bovine serum albumin (BSA; blocking solution, Winkler, Santiago, Chile) were used for 30 min each. Immunodetections were performed using primary anti-CCL3 (RD.MAB66252; R&D Systems, Inc., Minneapolis, MN, USA), anti-CCL21 (RD.AF457; R&D Systems, Inc., Minneapolis, MN, USA), anti-CD68 (ab125212, Abcam, Cambridge, UK), anti-B7-2 (SC-28347, Santa Cruz Biotechnology, Santa Cruz, CA, USA), anti-CD163 (SC-58965, Santa Cruz Biotechnology, Santa Cruz, CA, USA), anti-CD3 (Ab16669, Abcam, Cambridge, UK), and anti-MPO light chain/Myeloperoxidase (SC-390109, Santa Cruz Biotechnology, Santa Cruz, CA, USA) antibodies in blocking solution overnight at 4 °C. The immunosignals in IHC were visualized with the use of the ImmPRESS™ Excel Amplified HRP (peroxidase) Polymer Staining Kit (Vector Laboratories, Newark, CA, USA). Counterstaining of nuclei was performed by applying hematoxylin classic stain, followed by mounting the slides using Canada balsam Mounting medium (C1795, Merck, Darmstadt, Germany). Images were captured using a Bright-field microscope (Zeiss) and analyzed using color deconvolution with the Image J software Version 1.53t (NIH, Bethesda, MD, USA). For immunohistofluorescence (IHF) the samples were incubated with secondary antibodies Alexa 488 and 568 (1:250 dilution; Thermo Fisher Scientific, Waltham, MA, USA) for 60 min. Samples were incubated with DAPI (300 nM; Thermo Fisher Scientific, Waltham, MA, USA) for 10 min as a nuclei counterstain. To decrease tissue autofluorescence, 3% Sudan black B (*w*/*v* in 80% ethanol) stain was employed for 20 min. Finally, samples were washed in 1X PBS and mounted using a fluorescent mounting medium (S3023, Agilent-DAKO, Santa Clara, CA, USA). Images were captured using an epifluorescence microscope (Zeiss) and analyzed with Image J software Version 1.53t (NIH, Bethesda, MD, USA).

### 4.5. ELISA

The glomeruli were cultured ex vivo in Ham’s F-10 medium for 18 h. Then, this culture medium (conditioned medium) was collected, centrifuged at 800× *g*, and passed through a 0.45-μm filter. The glomerular levels of CCL2 (MBS2701125), CCL3 (MBS260259), CCL6 (MBS1604702), CCL21 (MBS269092), and CXCL9 (MBS2703909) in conditioned medium (CM) of ex vivo cultured glomeruli were quantified with the use of the commercial ELISA kit (MyBioSource, Southern California, San Diego, CA, USA) for each chemoattractant/chemokine according to the protocol provided by the manufacturer. The signals were then measured using a Synergy HTX microplate reader (Synergy HTX, BioTek, Winooski, VT, USA). The concentration of each chemoattractant/chemokine was normalized using H3 histone expression by western blot (Figure S3).

### 4.6. Macrophage In Vitro Cell Migration Assays

For the in vitro MΦ cell migration assay, we used 24-well plates with polycarbonate (PC) Boyden chamber (8 μm pore; Corning^®^, New York, NY, USA) as previously described [35]. We used the CM of the glomeruli from Ctrl, DN rats as a chemoattractant, and neutralizing antibodies against CCL2 (500 ng/mL; NBP1-07035; Novus Biologicals, Littleton, CO, USA), CCL3 (500 ng/mL; MAB66252; R&D Systems, Inc., Minneapolis, MN, USA), and CCL21 (500 ng/mL; AF457; R&D Systems, Inc., Minneapolis, MN, USA). The bottom of the transwell inserts were coated overnight with 15 μg/mL of bovine fibronectin. To isolate rat peripheral blood mononuclear cells (PBMCs), a protocol by flotation using a low density iodixanol (OptiPrepTM, Merck KGaA, Darmstadt, Germany; see Application Sheet C05 for details) barrier was used [35]. A total of 1 × 10^5^ rat MΦs were seeded into the top of the transwell inserts and 650 μL of CM was added in the bottom of the well. HAM-F10 and FBS (5%) were used as a chemoattractant negative and positive controls, respectively. Twelve hours later, MΦs in the bottom of the well were fixed with 70% ethanol for 10 min and stained using DAPI (300 nM) for 10 min. Five different quadrants were captured using 400× magnification in an epifluorescence microscope (Zeiss) and cells were counted using Image J software Version 1.53t (NIH, Bethesda, MD, USA).

### 4.7. Statistical Analysis

Graphs and statistical analyses were performed using GraphPad Prism 9 Software (Dotmatics, San Diego, CA, USA). Values are means ± standard deviation (SD), where n indicates the number of animals or times the assay was performed. Comparisons between two or more groups were conducted using the unpaired Student’s *t* test and two-way ANOVA, respectively. If the ANOVA demonstrated a significant interaction between variables, post hoc analyses were performed by the multiple comparison Bonferroni correction test. *p* ≤ 0.05 was considered statistically significant.

## 5. Conclusions

We concluded that the pharmacologic blockade of the A2BAR downregulate the transcriptional expression of genes and signaling pathways related to the immune response, decreases the protein expression and secretion of chemokines/chemoattractants, and diminishes the infiltration of monocytes/MΦs and pro-fibrotic M2 MΦ polarization in the glomeruli of DN rats, suggesting a new mechanism implicated in the anti-fibrotic effect of MRS1754.

## Figures and Tables

**Table 1:** Most significant immune system related pathways upregulated in the glomeruli of DN rats.

Pathway Name	Entities				Reactions	
	**Found**	**Ratio**	***p*-Value**	**FDR**	**Found**	**Ratio**
Interleukin-33 signaling	2/6	2.67 × 10^−4^	9.50 × 10^−4^	0.752	2/2	1.41 × 10^−4^
Chemokine receptors bind chemokines	4/102	0.005	0.007	0.752	4/19	0.001
Interleukin-10 signaling	5/175	0.008	0.01	0.752	1/15	0.001
Interleukin receptor SHC signaling	2/32	0.001	0.024	0.752	6/6	4.23 × 10^−4^
Terminal pathway of complement	1/9	4.01 × 10^−4^	0.064	0.752	4/5	3.52 × 10^−4^
Peptide ligand-binding receptors	12/515	0.023	0.089	0.752	12/83	0.006
CASP8 activity is inhibited	1/13	5.79 × 10^−4^	0.092	0.752	2/2	1.41 × 10^−4^

Analysis was performed using https://reactome.org (accessed on 5 June 2023). Abbreviations: SHC: Src homology and collagen; CASP8: caspase-8; FDR: false discovery rate.

**Table 2:** Most significant immune related pathways downregulated in the glomeruli of DN + MRS1754 rats.

Pathway Name	Entities				Reactions	
	**Found**	**Ratio**	***p*-Value**	**FDR**	**Found**	**Ratio**
Neutrophil degranulation	45/478	0.021	5.57 × 10^−11^	8.60 × 10^−8^	10/10	7.04 × 10^−4^
PD-1 signaling	6/34	0.002	6.86 × 10^−4^	0.446	2/5	3.52 × 10^−4^
IRAK deficiency (TRL2/4)	6/36	0.002	9.19 × 10^−4^	0.446	2/2	1.41 × 10^−4^
Interleukin-10 signaling	17/175	0.008	0.001	0.446	13/15	0.001
RUNX3 Regulates Immune Response and Cell Migration	4/19	8.46 × 10^−4^	0.003	0.799	2/5	3.52 × 10^−4^
MyD88 deficiency (TLR2/4)	6/46	0.002	0.003	0.799	2/2	1.41 × 10^−4^
Translocation of ZAP-70 to Immunological synapse	5/40	0.002	0.008	0.963	4/4	2.82 × 10^−4^
Cross-presentation of particulate exogenous antigens (phagosomes)	3/14	6.24 × 10^−4^	0.009	0.963	3/3	2.11 × 10^−4^
Immunoregulatory interactions between a Lymphoid and a non- Lymphoid cell	40/599	0.027	0.011	0.963	29/44	0.003
Phosphorylation of CD3 and TCR zeta chains	6/60	0.003	0.011	0.963	7/7	4.93 × 10^−4^
Regulation of Complement cascade	20/234	0.01	0.029	0.963	35/42	0.003
Complement cascade	21/260	0.012	0.03	0.963	52/72	0.005
Alternative complement activation	4/22	9.80 × 10^−4^	0.03	0.963	9/9	6.34 × 10^−4^
Chemokine receptors bind chemokines	10/102	0.005	0.038	0.963	9/19	0.001
Trafficking and processing of endosomal TLR	5/61	0.003	0.04	0.963	7/7	4.93 × 10^−4^

Analysis was performed using https://reactome.org (accessed on 5 June 2023). Abbreviations: PD-1: programmed death-1; IRAK: IL-1R-associated kinase; RUNX3: Runt-related transcription factor 3; MyD88: Myeloid differentiation primary response 88; TCR: T cell receptor; TLR: Toll-like receptor; FDR: false discovery rate.

## Data Availability

Data files are available upon request.

## Supplementary Materials

The supporting information can be downloaded at: https://www.mdpi.com/article/10.3390/ijms241310829/s1.
