# Supplementary material for: A2BAR Antagonism Decreases the Glomerular Expression and Secretion of Chemoattractants for Monocytes and the Pro-Fibrotic M2 Macrophages Polarization during Diabetic Nephropathy
Source: Int J Mol Sci. 2023 Jun 29;24(13):10829. doi: 10.3390/ijms241310829 (PMC10342004; doi:10.3390/ijms241310829)
Supplement: Supplementary file 1 [file ijms-24-10829-s001.zip › Supplementary Figures S1-S3.pdf]

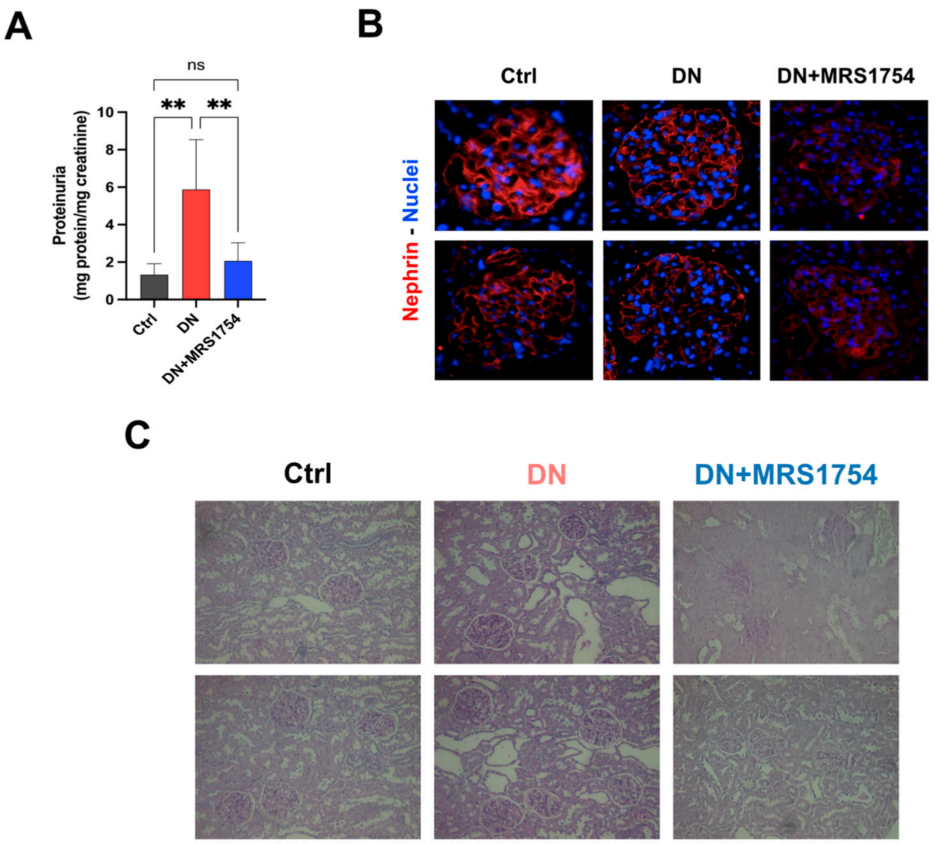

Figure S1. Validation of DN rat model treated with MRS1754. A) At week 12 post-STZ inoculation urine of non-diabetic (Ctrl), diabetic nephropathy (DN) and DN MRS1754-treated (DN+MRS1754) rats were collected using metabolic cages for 6 hours. Proteinuria was evaluated using an autoanalyzer CM250 (#1690007, Wiener Lab, Rosario, Argentina). Diabetic rats were treated with MRS1754 (0.5mg/kg/48 h i.p.) for eight weeks, four weeks after STZ inoculation. **B)** Immunohistofluorescence of the podocyte marker - nephrin in Ctrl, DN and DN+MRS1754 rats at week twelve post-STZ inoculation. DAPI (blue) was used as a counterstain. **C)** Periodic acid–Schiff (PAS) stain in kidneys of Ctrl, DN and DN+MRS1754 rats at week twelve post-STZ inoculation.

The graph represents the proteinuria (mean ± S.D). ** $p < 0.01$, between groups. n = 4-6 animals per group.

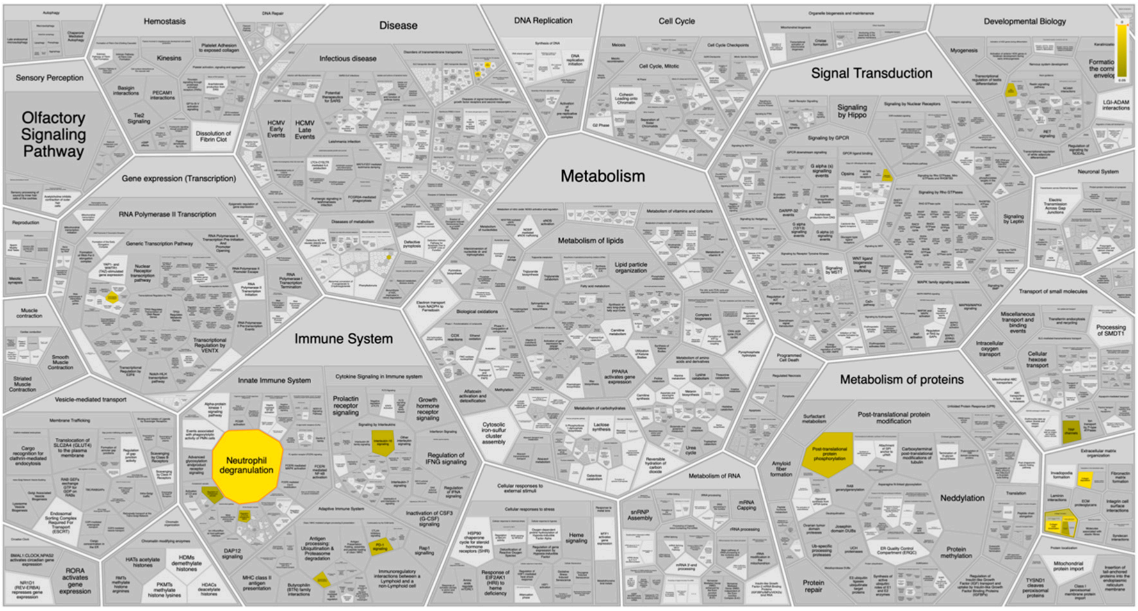

Figure S2. Genome-wide overview in glomeruli of DN+MRS1754 rats. Reactome pathways are arranged in a hierarchy. The color code denotes over-representation of that pathway in the input dataset. Light grey signifies pathways which are not significantly over-represented. Analysis was performed using https://reactome.org

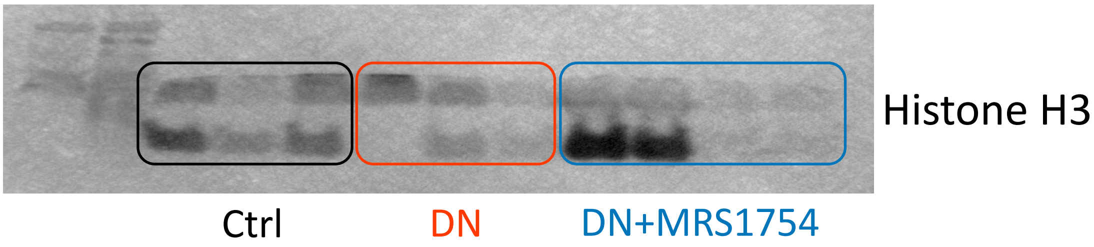

Figure S3. Histone H3 expression in glomeruli of DN+MRS1754 rats. Western blot of Histone H3 in the glomeruli of Ctrl (n=6), DN (n=6) and DN+MRS1754 (n=8). The Histone H3 expression was used to normalize the concentration of chemokines/chemoattractant measured by ELISA.
