# Supplementary material for: A2BAR Antagonism Decreases the Glomerular Expression and Secretion of Chemoattractants for Monocytes and the Pro-Fibrotic M2 Macrophages Polarization during Diabetic Nephropathy
Source: Int J Mol Sci. 2023 Jun 29;24(13):10829. doi: 10.3390/ijms241310829 (PMC10342004; doi:10.3390/ijms241310829)
Supplement: Supplementary file 1 [file ijms-24-10829-s001.zip › Table S1.pdf]

Table S1. List of upregulated transcripts related to the immune system in glomeruli of diabetic nephropathy rats treated with MRS1754.

Gene Symbol	Transcript	logFC	logCPM	p-value
Col18a1	NM_053489	1,31	9,45	0,05
Actn1	NM_031005	1,31	7,36	0,05
Synpo	NM_021695	1,31	10,38	0,05
Snta1	NM_001100901	1,33	5,88	0,05
Pdpn	NM_019358	1,33	8,05	0,05
B3galt2	NM_001109492	1,35	7,36	0,04
Aldh1a3	NM_153300	1,35	5,25	0,05
Cxadr	NM_053570	1,35	4,40	0,05
Fads1	NM_053445	1,36	5,99	0,04
Atp8b1	NM_001106140	1,36	7,37	0,04
Scx	NM_001130508	1,37	4,28	0,05
Slc7a7	NM_031341	1,42	7,14	0,03
Nptx2	NM_001034199	1,42	2,85	0,05
Hs3st3b1	NM_001191646	1,43	5,29	0,04
Ptprs	NM_019140	1,44	6,89	0,03
Unc13b	NM_001042579	1,44	4,68	0,04
Syt17	NM_138849	1,44	3,56	0,04
Pard6g	NM_001100973	1,45	3,02	0,04
Sema5a	NM_001107659	1,47	9,31	0,03
Nav2	NM_138529	1,47	6,79	0,03
Sema3e	NM_001106579	1,48	7,14	0,03
Lrrc4	NM_001037336	1,49	3,99	0,03
Itgb8	NM_001108726	1,49	4,56	0,03
Ren	NM_012642	1,50	4,21	0,03
Ptpro	NM_017336	1,51	10,62	0,02
Hhip	NM_001191817	1,53	8,10	0,02
Thbs1	NM_001013062	1,53	8,72	0,02
Igfbp2	NM_013122	1,57	8,28	0,02
Ptch1	NM_053566	1,57	7,69	0,02
Sema3g	NM_001100882	1,58	11,91	0,02
Gpm6b	NM_138846	1,59	4,61	0,02
B3gat1	NM_054003	1,60	3,82	0,02
L1cam	NM_017345	1,64	4,41	0,02
Sema3c	NM_001106578	1,64	6,87	0,01
Pak1	NM_017198	1,64	7,23	0,01
Adora2b	NM_017161	1,66	2,42	0,03
Fmod	NM_080698	1,70	2,88	0,02
Osr2	NM_001012118	1,70	2,78	0,02
C4a	NM_031504	1,72	4,90	0,01
Tmem37	NM_139095	1,73	3,72	0,01
Ptgfrn	NM_019243	1,75	6,18	0,01
Msh5	NM_212536	1,75	2,21	0,03
Akap12	NM_057103	1,77	1,68	0,03
Prrt2	NM_001276470	1,79	3,82	0,01
Slit1	NM_022953	1,80	2,85	0,02
Npr3	NM_012868	1,82	8,32	0,01
Ppp1r14c	NM_133425	1,83	1,72	0,03
Mgat5	NM_023095	1,83	8,50	0,01
Lbp	NM_017208	1,86	3,00	0,01

Robo3	NM_001108135	1,87	3,10	0,01
Rgs7	NM_019343	1,90	2,93	0,01
Pgm5	NM_001191953	1,92	2,84	0,01
Radil	NM_001037218	1,94	0,90	0,03
Slc2a10	NM_001108963	1,94	0,90	0,03
Tmem171	NM_001013903	1,95	1,81	0,02
Entpd2	NM_172030	1,96	2,64	0,01
Sost	NM_030584	1,99	1,31	0,03
Heyl	NM_001107977	2,00	3,71	0,01
Espl1	NM_001170602	2,00	2,06	0,01
Angptl2	NM_133569	2,02	7,59	0,00
Semg1	NM_012710	2,03	1,87	0,01
Zbtb16	NM_001013181	2,03	6,26	0,00
Col15a1	NM_001100535	2,07	3,34	0,00
Pla2g2a	NM_031598	2,14	1,05	0,02
Dusp4	NM_022199	2,15	1,42	0,02
Sorcs1	NM_001191563	2,17	6,19	0,00
Cd55	NM_022269	2,18	7,66	0,00
Tert	NM_053423	2,23	1,12	0,01
Megf6	NM_022955	2,26	5,07	0,00
Gps1	NM_001270939	2,27	0,63	0,02
Cldn1	NM_031699	2,29	5,42	0,00
Lcn2	NM_130741	2,30	3,43	0,00
Padi2	NM_017226	2,32	5,20	0,00
Mmp9	NM_031055	2,39	8,13	0,00
Kap	NM_052802	2,44	5,80	0,00
Dyrk3	NM_001024767	2,58	0,05	0,04
Siglec10	NM_001106250	2,58	0,05	0,04
Aire	NM_001106379	2,58	0,05	0,04
Pbk	NM_001079937	2,61	0,89	0,01
Rhbdl2	NM_001106684	2,61	0,89	0,01
Trim46	NM_001107691	2,61	0,89	0,01
Mgat4a	NM_001012225	2,68	1,84	0,00
Cyp27b1	NM_053763	2,76	1,52	0,00
Galnt13	NM_199106	2,76	1,52	0,00
Myom2	NM_001169141	2,81	5,51	0,00
Depdc1	NM_001305205	2,89	1,11	0,00
Nptx1	NM_153735	2,90	4,26	0,00
Arl14	NR_131117	2,94	0,31	0,01
Tenm4	NM_001191628	2,94	4,43	0,00
Tox2	NM_199392	3,05	1,24	0,00
Saa4	NM_001009478	3,16	0,48	0,01
Itm2a	NM_001025712	3,23	0,53	0,00
RT1-CE1	NM_001008832	3,23	1,92	0,00
Fgb	NM_020071	3,38	3,20	0,00
Col2a1	NM_012929	3,45	3,05	0,00
RT1-CE5	NM_001033986	3,69	2,71	0,00
Ubd	NM_053299	3,85	1,04	0,00
Eda	NM_001305243	4,08	1,24	0,00
Adora1	NM_017155	4,08	1,24	0,00
Arpp21	NM_001135045	4,15	1,30	0,00
Dntt	NM_001012461	5,95	-0,68	0,02
Retnlb	NM_001024281	5,95	-0,68	0,02

Asb4	NM_001024318	5,95	-0,68	0,02
PCOLCE2	NM_001127640	5,95	-0,68	0,02
Atp13a5	NM_001191657	5,95	-0,68	0,02
Msc	NM_001191754	5,95	-0,68	0,02
Col26a1	NM_001271268	5,95	-0,68	0,02
Mylpf	NM_012605	5,95	-0,68	0,02
Smad2	NM_019191	5,95	-0,68	0,02
Shank2	NM_133441	5,95	-0,68	0,02
Krt17	NM_212545	5,95	-0,68	0,02
Prrt2	NR_077057	5,95	-0,68	0,02
Sarm1	NM_001105817	6,26	-0,46	0,01
Pla2g5	NM_017174	6,26	-0,46	0,01
Sh2d1a	NM_001109313	6,52	-0,27	0,01
Nkx6-3	NM_001109455	6,52	-0,27	0,01
Prss38	NM_001304375	6,52	-0,27	0,01
Adcyap1	NM_016989	6,52	-0,27	0,01
Il1rap	NM_001167840	6,74	-0,10	0,00
Cav1	NM_133651	6,74	-0,10	0,00
Shc4	NM_001191065	6,93	0,05	0,00
Prss16	NM_001033710	7,39	0,42	0,00

Analysis was performed using https://reactome.org
