# Supplementary material for: A2BAR Antagonism Decreases the Glomerular Expression and Secretion of Chemoattractants for Monocytes and the Pro-Fibrotic M2 Macrophages Polarization during Diabetic Nephropathy
Source: Int J Mol Sci. 2023 Jun 29;24(13):10829. doi: 10.3390/ijms241310829 (PMC10342004; doi:10.3390/ijms241310829)
Supplement: Supplementary file 1 [file ijms-24-10829-s001.zip › Table S3.pdf]

Table S3. Most significant pathways dysregulated in the DM+MRS1754 glomeruli.

Term or signaling pathway	Count	P-Value	Fold Enrichment	FDR
Metabolic pathways	87	0,015	1,3	0,07
Cell adhesion molecules	29	9,2E-10	3,9	0,000000056
Tuberculosis	29	1,8E-09	3,8	0,000000088
Staphylococcus aureus infection	27	5E-14	6,3	1,2E-11
Phagosome*	26	0,00000033	3,3	0,000011
Cytokine-cytokine receptor interaction*	23	0,0028	2	0,021
Coronavirus disease - COVID-19	23	0,018	1,7	0,082
Complement and coagulation cascades*	22	5,3E-11	5,9	6,5E-09
Chemokine signaling pathway*	22	0,000035	2,8	0,0007
Neutrophil extracellular trap formation*	21	0,00013	2,6	0,002
Human T-cell leukemia virus 1 infection	21	0,0053	2	0,031
Natural killer cell mediated cytotoxicity*	20	0,000000033	4,7	0,0000013
Leishmaniasis	19	6,7E-10	6,2	0,000000054
Focal adhesion	19	0,0024	2,2	0,02
Proteoglycans in cancer	19	0,0035	2,1	0,025
Rap1 signaling pathway	19	0,0048	2,1	0,03
Bile secretion	18	0,00000043	4,4	0,000013
Osteoclast differentiation	18	0,000015	3,4	0,00034
Hematopoietic cell lineage	17	0,0000012	4,4	0,000032
Systemic lupus erythematosus	17	0,000047	3,3	0,00085
Axon guidance	17	0,005	2,2	0,03
Regulation of actin cytoskeleton	17	0,031	1,8	0,12
Calcium signaling pathway	17	0,051	1,7	0,18

Viral myocarditis	16	0,0000028	4,4	0,000069
Leukocyte transendothelial migration*	16	0,00014	3,2	0,002
Platelet activation	16	0,00032	2,9	0,004
cAMP signaling pathway	16	0,047	1,7	0,17
Inflammatory mediator regulation of TRP channels*	15	0,0004	3	0,0047
Lipid and atherosclerosis	15	0,067	1,7	0,22
Th17 cell differentiation*	14	0,0005	3,1	0,0053
Vascular smooth muscle contraction	14	0,0068	2,3	0,038
B cell receptor signaling pathway*	13	0,0001	3,9	0,0016
Antigen processing and presentation*	13	0,00033	3,5	0,004
Amoebiasis	13	0,00081	3,1	0,0079
Protein digestion and absorption	13	0,0015	2,9	0,014
Chagas disease	13	0,0017	2,9	0,014
Inflammatory bowel disease	12	0,000049	4,6	0,00085
Fc gamma R-mediated phagocytosis*	12	0,0025	2,9	0,02
C-type lectin receptor signaling pathway*	12	0,0093	2,5	0,049
Alcoholic liver disease	12	0,031	2,1	0,12
Graft-versus-host disease	11	0,00025	4,2	0,0033
Pertussis	11	0,0011	3,5	0,01
Arachidonic acid metabolism	11	0,0026	3,1	0,02
Viral protein interaction with cytokine and cytokine receptor	11	0,0034	3	0,024
Rheumatoid arthritis	11	0,0037	3	0,025
Th1 and Th2 cell differentiation*	11	0,0048	2,9	0,03
Toll-like receptor signaling pathway*	11	0,0065	2,7	0,037
Toxoplasmosis	11	0,021	2,3	0,09
Relaxin signaling pathway	11	0,049	2	0,18
Yersinia infection	11	0,066	1,9	0,22

Type I diabetes mellitus	10	0,0027	3,4	0,02
Chemical carcinogenesis - DNA adducts	10	0,0052	3	0,031
PPAR signaling pathway	10	0,011	2,7	0,058
ECM-receptor interaction	10	0,012	2,7	0,061
Dilated cardiomyopathy	10	0,02	2,5	0,085
Parathyroid hormone synthesis, secretion and action	10	0,037	2,2	0,14
Pancreatic secretion	10	0,037	2,2	0,14
Serotonergic synapse	10	0,083	1,9	0,26
Intestinal immune network for IgA production	9	0,00042	4,8	0,0047
Allograft rejection	9	0,0049	3,4	0,03
Autoimmune thyroid disease	9	0,011	2,9	0,057
Thyroid hormone synthesis	9	0,013	2,9	0,062
Metabolism of xenobiotics by cytochrome P450	9	0,016	2,7	0,075
Salivary secretion	9	0,023	2,6	0,096
Insulin secretion	9	0,03	2,5	0,12
Drug metabolism - other enzymes	9	0,055	2,2	0,19
AGE-RAGE signaling pathway in diabetic complications	9	0,069	2,1	0,22
Malaria	8	0,0089	3,4	0,048
Retinol metabolism	8	0,077	2,2	0,25
Asthma	7	0,00068	6,2	0,0069
Cysteine and methionine metabolism	7	0,02	3,2	0,085
Regulation of lipolysis in adipocytes	7	0,033	2,9	0,13
Fc epsilon RI signaling pathway*	7	0,064	2,5	0,22
Primary immunodeficiency	6	0,018	3,9	0,082
Carbohydrate digestion and absorption	6	0,047	3	0,17
Mineral absorption	6	0,098	2,4	0,29
Biosynthesis of unsaturated fatty acids	5	0,057	3,4	0,2

Linoleic acid metabolism	5	0,092	2,9	0,28
African trypanosomiasis	5	0,092	2,9	0,28
Pantothenate and CoA biosynthesis	4	0,059	4,4	0,2
alpha-Linolenic acid metabolism	4	0,082	3,9	0,26

Analysis was performed with KEGG using https://david.ncifcrf.gov/

*** Term or pathway related to the immune system**
